# Polyaniline/Ti_3_C_2_ MXene Composites with Artificial 3D Biomimetic Surface Structure of Natural Macaw Feather Applied for Anticorrosion Coatings

**DOI:** 10.3390/biomimetics10070465

**Published:** 2025-07-15

**Authors:** Chen-Cheng Chien, Yu-Hsuan Liu, Kun-Hao Luo, Ting-Yun Liu, Yi-Ting Kao, Shih-Harn Yang, Jui-Ming Yeh

**Affiliations:** 1 Department of Chemistry, Chung Yuan Christian University, Chung Li District‚ Taoyuan City 32023, Taiwan; 2 Department of Program in Indigenous Culture and Design, Chung Yuan Christian University, Chung Li District‚ Taoyuan City 32023, Taiwan

**Keywords:** PANI, MXene, composite, biomimetic, corrosion

## Abstract

In this paper, a series of polyaniline (PANI)/Ti_3_C_2_ MXene composites (PMCs) with a biomimetic structure were prepared and employed as an anticorrosion coating application. First, the PANI was synthesized by oxidative polymerization with ammonium persulfate as the oxidant. Then, 2D Ti_3_C_2_ MXene nanosheets were prepared by treating the Ti_3_AlC_2_ using the optimized minimally intensive layer delamination (MILD) method, followed by characterization via XRD and SEM. Subsequently, the PMC was prepared by the oxidative polymerization of aniline monomers in the presence of Ti_3_C_2_ MXene nanosheets, followed by characterization via FTIR, XRD, SEM, TEM, CV, and UV–Visible. Eventually, the PMC coatings with the artificial biomimetic surface structure of a macaw feather were prepared by the nano-casting technique. The corrosion resistance of the PMC coatings, evaluated via Tafel polarization and Nyquist impedance measurements, shows that increasing the MXene loading up to 5 wt % shifts the corrosion potential (E_corr_) on steel from −588 mV to −356 mV vs. SCE, reduces the corrosion current density (I_corr_) from 1.09 µA/cm^2^ to 0.035 µA/cm^2^, and raises the impedance modulus at 0.01 Hz from 67 kΩ to 3794 kΩ. When structured with the hierarchical feather topography, the PMC coating (Bio-PA-MX-5) further advances the E_corr_ to +103.6 mV, lowers the I_corr_ to 7.22 × 10^−4^ µA/cm^2^, and boosts the impedance to 96,875 kΩ. Compared to neat coatings without biomimetic structuring, those with engineered biomimetic surfaces showed significantly improved corrosion protection performance. These enhancements arise from three synergistic mechanisms: (i) polyaniline’s redox catalysis accelerates the formation of a dense passive oxide layer; (ii) MXene nanosheets create a tortuous gas barrier that cuts the oxygen permeability from 11.3 Barrer to 0.9 Barrer; and (iii) the biomimetic surface traps air pockets, raising the water contact angle from 87° to 135°. This integrated approach delivers one of the highest combined corrosion potentials and impedance values reported for thin-film coatings, pointing to a general strategy for durable steel protection.

## 1. Introduction

Corrosion is a widespread and damaging issue that affects numerous technological sectors, such as the marine industry, automotive industry, rail transportation, and construction. Worldwide, the economic impact of corrosion exceeds USD 4 trillion annually [1]. Enhancing the corrosion resistance of metals has been pursued through strategies like cathodic protection [2], superhydrophobic surfaces [3,4], and organic coatings [5]. Among the various corrosion protection methods, the use of epoxy-based organic coatings is the most widely adopted for safeguarding steel structures, owing to their ease of application, high effectiveness, and cost-efficiency. While epoxy coatings provide a physical barrier protecting metal surfaces from corrosive environments, permeation by aggressive species such as H_2_O, O_2_, Cl^−^, and H^+^ may occur under severe corrosive exposure. Such permeation results in blistering, delamination, and coating degradation, ultimately inducing corrosion of the steel substrate [6].

Significant research has been directed toward 2D lamellar materials such as graphene [7], graphene oxide [8], h-BN [9], and clay [10] for corrosion protection, motivated by their non-toxic characteristics and superior barrier performance. Recently, two-dimensional (2D) MXene laminates have attracted significant research interest due to their hybrid ceramic–metallic properties, including high strength, elastic modulus, chemical stability, exceptional electrical and thermal conductivity, and mechanical workability [11,12,13]. The research work in terms of MXene-based polyaniline composite anticorrosion coatings has been published by several groups [14,15,16,17,18,19,20].

Biomimetics constitutes an interdisciplinary domain dedicated to deciphering the functions, structures, and governing principles of biological systems. Natural materials achieve exceptional precision and efficiency through precisely engineered hierarchical organization spanning nanoscale to macroscale dimensions, fulfilling specialized functional requirements. The multifunctionality of natural materials, biological systems, and surfaces arises from the complex integration of their physical properties, chemical composition, morphological features, surface topography, texture, and other intrinsic attributes. This integrated synergy enables exceptional multifunctionality, reflecting the intricate complexity of biological design. Numerous natural phenomena have garnered significant attention for their potential applications across various industrial sectors, such as super-hydrophobicity, superoleophobicity [21], high/reversible adhesion [22], refection/antireflection [23], self-cleaning, friction lessening [24], energy conversion, thermal insulation, self-healing, and others [25]. Yeh et al. reported that polymer coatings with a biomimetic surface structure that was natural lotus-leaf-like may effectively enhance the corrosion protection of metallic substrates [26,27,28,29]. On the other hand, Yeh et al. also reported that polymer coatings with a biomimetic surface structure of a natural feather may also effectively promote the corrosion protection of metallic substrates [30]. Moreover, the incorporation of two-dimensional materials such as nano-clay [31], ZnO [32], mesoporous silica [33], or biomass carbon [34] in polymer coatings with biomimetic surface structures may further boost the corresponding corrosion protection performance. However, a polymer coating with a biomimetic surface structure containing MXene platelets has seldom been mentioned.

To address the persistent challenge of protecting steel in aggressive environments, the authors have developed a single coating system that integrates three complementary corrosion-resistance strategies within a biomimetic PANI/MXene composite. First, the intrinsically redox-active polyaniline matrix catalyzes the rapid formation of a uniform iron-oxide passive film, enhancing metal passivation. Second, embedded Ti_3_C_2_ MXene nanosheets create a highly tortuous diffusion path that dramatically reduces the oxygen and moisture permeability. Third, an imprinted hierarchical macaw-feather surface traps air pockets and repels aqueous electrolytes, conferring hydrophobicity without the need for low-surface-energy additives. By combining electrocatalytic passivation, two-dimensional barrier reinforcement, and three-dimensional surface structuring in one formulation, our coating outperforms conventional single-mode systems and offers a versatile platform for long-term steel protection.

## 2. Materials and Methods

### 2.1. Chemicals

Aniline (>99%, Alfa Aesar, Ward Hill, MA, USA) was doubly distilled prior to use. Titanium aluminum carbide (Ti_3_AlC_2_) (≥90%, ≤40 µm, Sigma-Aldrich, St. Louis, MO, USA), hydrochloric acid (Sigma-Aldrich) and ammonia solution (Sigma-Aldrich), lithium fluoride (98.5%, Thermo Scientific, Waltham, MA, USA), calcium hydroxide (≥98%, Thermo Scientific), ammonium peroxydisulfate (APS, (NH_4_)_2_S_2_O_8_, 98%, Showa, Tokyo, Japan), N-methyl-2-pyrrolidone (>99%, Sigma-Aldrich), hydrazine diazane (≥98%, Alfa Aesar), and sodium chloride (J. T. Baker, Phillipsburg, NJ, USA) polydimethylsiloxane (PDMS, Sil-More Industrial Ltd., New Taipei City, Taiwan) were all used as received without further purification.

### 2.2. Instruments and Methods

The FT-IR spectra of materials and samples were collected using an FT-IR spectrometer (Bio-Rad FTS-7, Bio-Rad Laboratories, Hercules, CA, USA) at room temperature. The attenuated total reflectance FT-IR (ATR-FTIR) spectra were collected at a resolution of 4.0 cm^−1^. X-ray diffraction (XRD) patterns of the as-prepared materials were recorded using a Bruker D8 Advance Eco diffractometer (formerly Philips X’pert Pro platform, Malvern Panalytical, Almelo, The Netherlands) with Cu Ka (λl = 0.15418 nm). The morphological structure of the as-prepared materials was observed under a field-emission scanning electron microscopy (FE-SEM) system (JEOL JSM-7600F, JEOL Ltd., Tokyo, Japan) and transmission electron microscope (TEM, JEOL JEM-2000FX, JEOL Ltd., Tokyo, Japan), respectively. An ultraviolet–visible spectrophotometer (UV-Vis, Jasco V-750, JASCO Corporation, Tokyo, Japan) and cyclic voltammetry (VoltaLab 50 (PST050), Radiometer Analytical, Lyon, France) were used to monitor the redox capability of the as-prepared materials. The micropore size and surface area of the as-prepared samples were calculated from N_2_ adsorption–desorption isotherms measured at 77 K using Brunauer–Emmett–Teller (BET) (Micromeritics Instrument Corp, Norcross, GA, USA) analysis on a Micromeritics ASAP 2020 instrument. The surface wettability of the as-prepared materials was characterized by water contact angle (WCA) measurements using a First Ten Angstroms FTA-125 goniometer. After depositing water droplets (~4 μL) onto the sample surface, the contact angle (CA) was calculated as the average of five measurements taken at different locations. The gas barrier properties of the as-prepared membrane materials were evaluated using a gas permeability analyzer (GPA, GTR-31). The electrochemical corrosion characteristics of the coating samples were assessed using a VoltaLab 40 potentiostat/galvanostat in a standard three-electrode configuration: two graphite rods served as the counter electrode and a saturated calomel electrode (SCE) as the reference electrode. Electrochemical impedance spectroscopy (EIS) measurements were subsequently performed on the as-prepared materials using an AutoLab PGSTAT302N potentiostat/galvanostat workstation. All polarization experiments were performed below water-splitting limits; no gas evolution was detected, and the counter-electrode potential remained stable throughout each run.

### 2.3. Preparation of Ti_3_C_2_ MXene Nanosheets

**Scheme 1:**
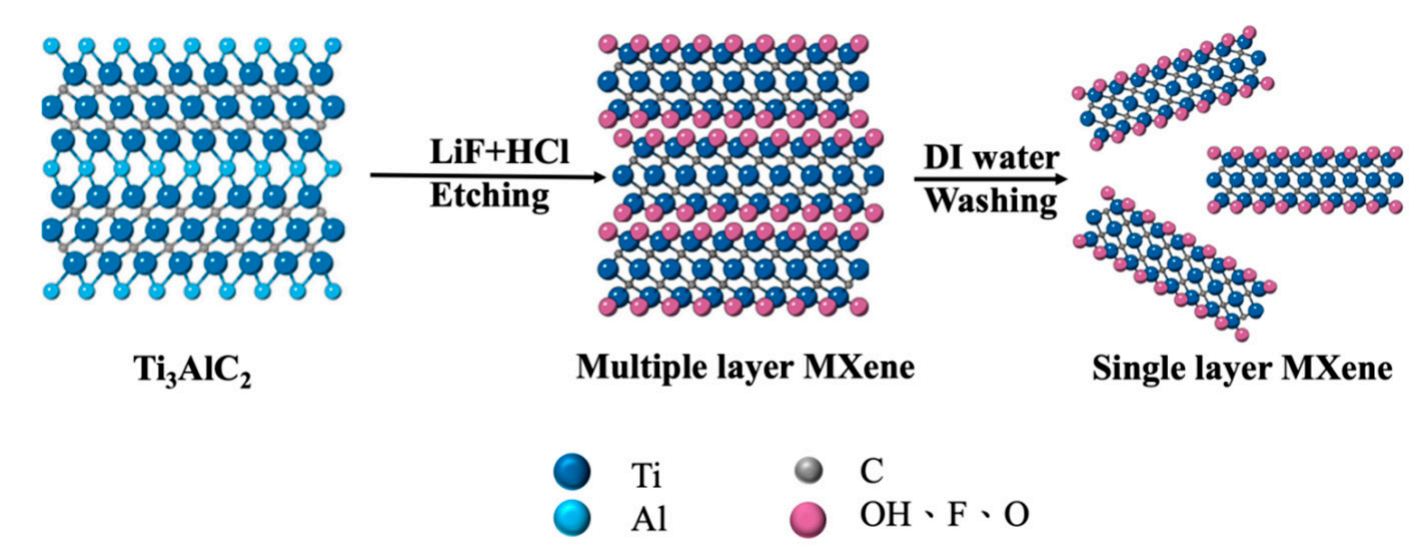
Schematic representation of the preparation of Ti_3_C_2_ MXene nanosheets by the minimally intensive layer delamination (MILD) method.

MXene was synthesized through the optimized Minimally Intensive Layer Delamination (MILD) method, which enables effective exfoliation while preserving the structural integrity of the material (Scheme 1) [14,15,16,17,18,19,20]. As a representative procedure for the preparation of MXene nanosheets, 1.0 g of lithium fluoride (LiF) was initially dissolved in 20 mL of a 9.0 M hydrochloric acid (HCl) aqueous solution within a polytetrafluoroethylene (PTFE) beaker, hereafter referred to as Beaker A. The solution in beaker A was under magnetic stirring for 15 min until the LiF powder in beaker A was entirely dissolved. Subsequently, 1 g of Ti_3_AlC_2_ powder was slowly added into a PTFE beaker, and the powder was etched by acid at 45 °C for 48 h. Moreover, the mixing solution in beaker A was treated by centrifugation, followed by pouring off the upper acidic liquid of beaker A, followed by washing and shaking the residue with DI water and repeating the centrifugation several times until the pH value of the pour-off upper acidic liquid of beaker A reached ~5. An excess amount of DI water was introduced into the previous product under ultrasonic shaking for 3 h, followed by centrifuging. Subsequently, a single layer of Ti_3_C_2_ MXene nanosheets was found in the upper layer of aqueous solution, and multiple-layers of Ti_3_C_2_ MXene nanosheets were found in the lower layer of precipitate.

### 2.4. Synthesis of Polyaniline (PANI)

As a representative procedure to prepare PANI [10], 1.488 g of distilled aniline monomers were introduced into 300 mL of 1.0 M HCl aqueous solution and kept at ~0 °C under magnetic stirring for 2 h (denoted beaker A). On the other hand, 9.12 g of ammonium persulfate (APS) functioning as the oxidant was dissolved in 50 mL of a 1.0 M HCl aqueous solution and was kept in an ice bath under magnetic stirring (beaker B). Subsequently, the solution in beaker B was slowly added dropwise to the solution of beaker A, followed by magnetic stirring for 4 h. The as-prepared PANI obtained after suction filtration underwent washing with an excess of 1.0 M aqueous ammonium hydroxide solution, followed by vacuum drying at 60 °C for 24 h. The final product yield was estimated at ~30 wt %.

### 2.5. Preparation of PA-MX-5

**Scheme 2:**
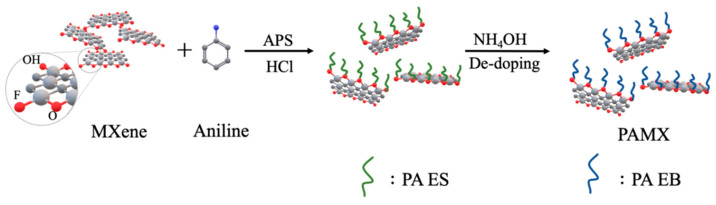
Schematic illustration of the process for the synthesis of PANI/Ti_3_C_2_ MXene composites by in-situ oxidative polymerization of aniline monomers in the presence of Ti_3_C_2_ MXene nanosheets.

As shown in Scheme 2, a representative procedure to prepare PA-MX-5 [15], first, 2.98 g of aniline monomers were introduced into 300 mL of 1.0 M HCl kept at 0 °C (denoted by beaker A). Subsequently, 0.149 g of the as-prepared MXene was added into beaker A under magnetic stirring for 2 h. On the other hand, 1.8254 g of APS, functioning as the oxidant, was introduced into 20 mL of 1.0 M HCl maintained at 0 °C (denoted by beaker B). Subsequently, the solution in beaker B was added drop-wise into the solution of beaker A under magnetic stirring for 4 h. After suction filtration, the resulting PA-MX-5 material was washed with an excess of 1.0 M NH_4_OH aqueous solution, followed by vacuum drying at 60 °C for 24 h.

### 2.6. Preparation of Free-Standing Membranes of PANI and Its Composites and Corresponding Barrier Property Measurements

Free-standing membranes of neat PANI and Ti_3_C_2_ MXene-based PANI composites were fabricated to evaluate their O_2_ permeability under high-pressure differential conditions. For composite preparation, a specified amount of PANI was dissolved in N-methyl-2-pyrrolidone (NMP), followed by the incorporation of 1, 3, and 5 wt % Ti_3_C_2_ MXene nanosheets under continuous magnetic stirring for 24 h. The prepared solution/dispersion was cast onto a substrate, typically a microscope glass slide, and dried under ambient conditions at 50 °C in a fume hood for 24 h. After complete drying, the coated substrate was soaked in deionized water for 24 h to release the free-standing PANI and composite membranes. Each membrane was then cut into a circular film with a diameter of 6 cm and a thickness of approximately 60 μm. Oxygen permeability measurements of the free-standing membranes were performed using a Yanaco GTR-10 gas permeability analyzer from Yanaco Analytical Systems Co., Kyoto, Japan. The permeability coefficient, denoted as *P*, was calculated employing Equation (1) [35]:

(1) P=1p1−p2×qtA,

where *P* represents the oxygen permeability coefficient in units of [cm^3^(STP), cm/cm^2^, s, cmHg)], *q*/*t* denotes the volumetric flow rate of permeated gas in [cm^3^(STP)/s], the thickness of the free-standing membrane in cm, *A* corresponds to the effective film area in cm^2^, with *P*_1_ and *P*_2_ indicating the pressures in cmHg on the high-pressure and low-pressure sides of the membrane, respectively. The measurement was conducted at 30 °C using carrier gas and air as the target gas in the GPA. The oxygen transmission rate (OTR) was quantified using gas chromatography, and the air permeability was derived to assess the barrier performance of each coating.

### 2.7. Preparation of PDMS Negative Template [26,27,28,29,30,31,32,33,34]

First, the natural template of macaw feathers obtained from a pasture in Taiwan was cut into appropriate size, followed by sticking it onto the Teflon board with 3M double-sided tape. Subsequently, the commercial compound of Sylgard 184A and Sylgard 184B was mixed homogeneously at a weight ratio of 10:1 in a beaker under magnetic stirring, followed by pouring onto the previous Teflon plate on which the natural feather of macaw was attached. After degassing treatment under vacuum, the final mixing product was placed in an oven and heated up to 80 °C for 8 h to complete the thermal cross-linking reaction of PDMS. Eventually, the natural feather was detached from the PDMS, and a final artificial PDMS negative template of the macaw feather structure was obtained.

### 2.8. Preparation of Coatings Without/with Biomimetic Structure of Macaw Feather

#### 2.8.1. Fabrication of PANI and PA-MX-5 Film

As a representative procedure to prepare the coating, first, 0.4 g of PANI and PA-MX-5 fine powder was dissolved in 19.6 mL of NMP, individually, under magnetic stirring to obtain both 2 wt % of PANI and PA-MX solution. Subsequently, the as-prepared solutions were cast onto the cold-rolled steel (CRS) electrode, respectively, followed by evaporating the solvent by heating in a fume hood. The CRS electrode coated with PANI and PA-MX-5 was obtained with ~80 μm of coating thickness.

#### 2.8.2. PANI and PA-MX with 3D Biomimetic Surface Structure of Macaw Feather

**Scheme 3:**
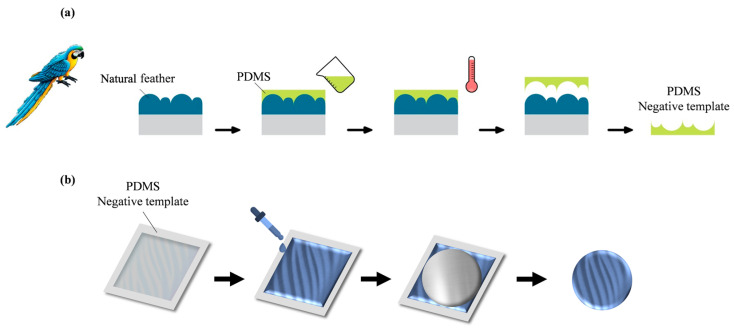
(**a**) Schematic representation of the preparation of biomimetic PDMS negative soft template of macaw feather. (**b**) Illustration of the preparation of PANI and its composite coatings with 3D biomimetic surface structure of macaw feather.

In Scheme 3, as a representative procedure to prepare the coating with the 3D biomimetic structure of a feather, the solutions of PA and PA-MX-5 were poured onto the surface of the as-prepared PDMS negative template, followed by heating to evaporate the partial organic solvent of the solutions. Subsequently, the CRS electrode was placed on the surface of the PDMS negative template coated with the solutions of PA and PA-MX-5. The heating progress upon the previous sample was maintained until all the organic solvent was completely evaporated, and the as-prepared PDMS template was fully cured. Finally, the CRS electrode with artificial PANI and PA-MX-5 coatings with the biomimetic surface structure of a natural macaw feather were obtained after carefully detaching the negative template of PDMS (denoted by Bio-PANI and Bio-PA-MX-5, respectively).

### 2.9. Electrochemical Corrosion Measurements

A predetermined amount of PANI was dissolved in NMP with varying Ti_3_C_2_ MXene nanosheets with loadings of 1, 3, and 5 wt %. The mixture was under magnetic stirring for 24 h. The resulting solutions were then carefully applied dropwise onto cold-rolled steel (CRS) electrodes and subsequently heated at 50 °C for 24 h, forming coatings with an approximate thickness of ~80 μm. Both uncoated and coated electrodes were mounted on the working electrode to ensure that only the coated surface was in direct contact with the electrolyte solution. To prevent side interactions, the edges of the electrodes were sealed with super-fast epoxy cement (SPAR). All electrochemical experiments were conducted at ambient temperature, with each experiment repeated at least three times for reproducibility. The electrolyte solution consisted of 3.5 wt % NaCl (aq). The open circuit potential (OCP), representing the equilibrium state of the system, was recorded as the corrosion potential (E_corr_, in V vs. SCE). The polarization resistance (R_p_, in Ω/cm^2^) was evaluated by altering the applied potential from 550 mV below to 550 mV above the E_corr_ at a scanning rate of 1000 mV/min, while observing the resulting current variations. The *R_p_* value was obtained from the slope of the potential–current relationship. Tafel plots were generated by scanning the potential within the same range, and the corrosion current density (i_corr_) was determined by linear extrapolation through the cathodic or anodic branch at E_corr_.

On the other hand, electrochemical impedance spectroscopy (EIS) probes the interfacial properties of CRS electrodes by applying an AC perturbation and measuring the complex impedance (Z), which can be expressed as follows [36]:

(2) $$Z(\omega )=Z^{\prime }+jZ\prime \prime =R_{s}+\frac{R_{ct}}{1+{\left(R_{ct}C_{dl}\omega \right)}^{2}}-j\frac{R_{ct}^{2}C_{dl}\omega }{{1+(R_{ct}C_{dl}\omega )}^{2}}.$$

**Figure 1:**
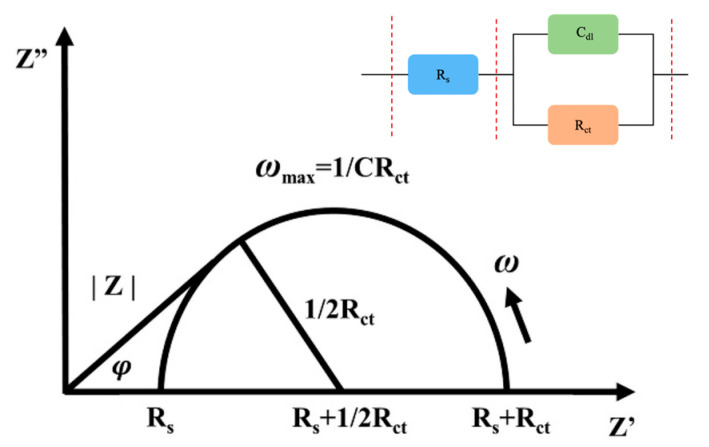
Equivalent circuit for fitting EIS, Nyquist plots, and fitting curves.

In our equivalent-circuit model, the solution resistance (*R_s_*) is in series with a parallel combination of the charge-transfer resistance (*R_ct_*) and the double-layer capacitance (*C_dl_*). Applying Kirchhoff’s laws to this *Rs* − [*R_ct_*‖*C_dl_*]. At high frequency (*ω* → ∞), *Z* ≈ *R_s_*, while at low frequency (*ω* → 0), *Z* ≈ *R_s_* + *R_ct_*; so, the diameter of the Nyquist semicircle directly corresponds to *R_ct_* [28,29]. A larger *R_ct_* therefore indicates a slower corrosion rate [30,31]. The equivalent circuit used to extract these parameters—*R_s_* in series with (*R_ct_*‖*CPE*)—is shown in Figure 1.

## 3. Results and Discussion

**Figure 2:**
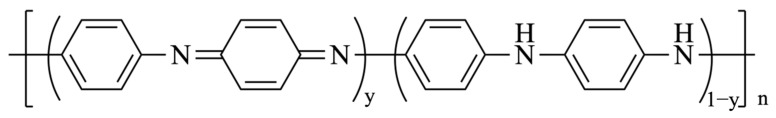
Polyaniline general formula.

The base forms of polyaniline prepared from oxidative polymerization can be schematically represented by the general formula in Figure 2 [10].

Here, the y value ranges from 1 for the fully oxidized polymer (so-called pernigr-aniline) to 0.5 for the half-oxidized polymer (emeraldine) and to 0 for the fully reduced polymer (leuco-emeraldine).

### 3.1. Identification of MXene

#### 3.1.1. XRD Analysis [14,15,16,17,18,19,20]

**Figure 3:**
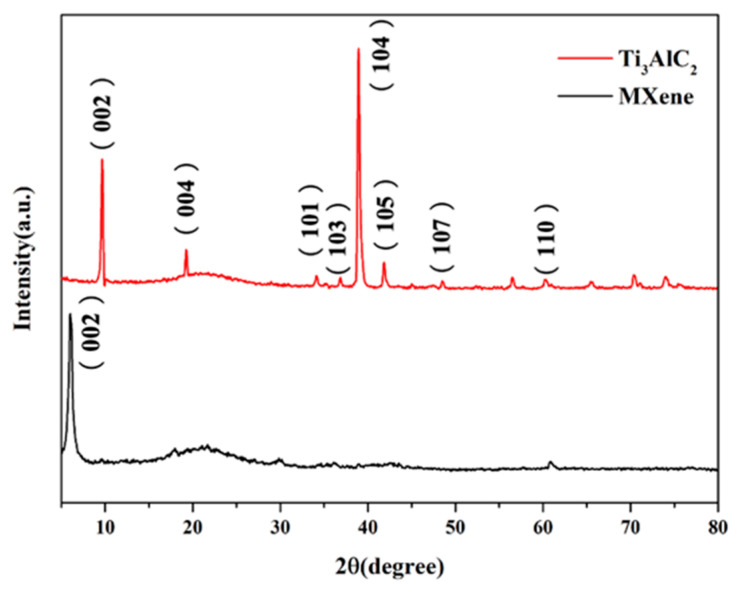
XRD patterns of Ti_3_AlC_2_ and Ti_3_C_2_ MXene nanosheets prepared by MILD method.

In the un-etched phase of Ti_3_AlC_2_, distinct characteristic peaks were observed at 2θ angles of 9.68°, 19.22°, and 38.94°, corresponding to the (002), (004), and (104) planes, respectively, as shown in Figure 3. After MILD etching, the (004) and (104) peaks almost vanish, demonstrating that the Al layers have been selectively removed, and the structure has been delaminated into Ti_3_C_2_ MXene. Meanwhile, the (002) peak shifts from 9.68° to ~6°, corresponding via Bragg’s law to an expansion of the interlayer spacing from 9.13 Å to 14.72 Å in line with the successful intercalation of water and Li^+^ during delamination, which promotes better exfoliation and a higher surface area for composite formation. The disappearance of higher-order peaks coupled with peak broadening also indicates a reduced stacking order, which is beneficial for subsequent PANI growth on individual MXene sheets.

#### 3.1.2. SEM Analysis [14,15,16,17,18,19,20]

**Figure 4:**
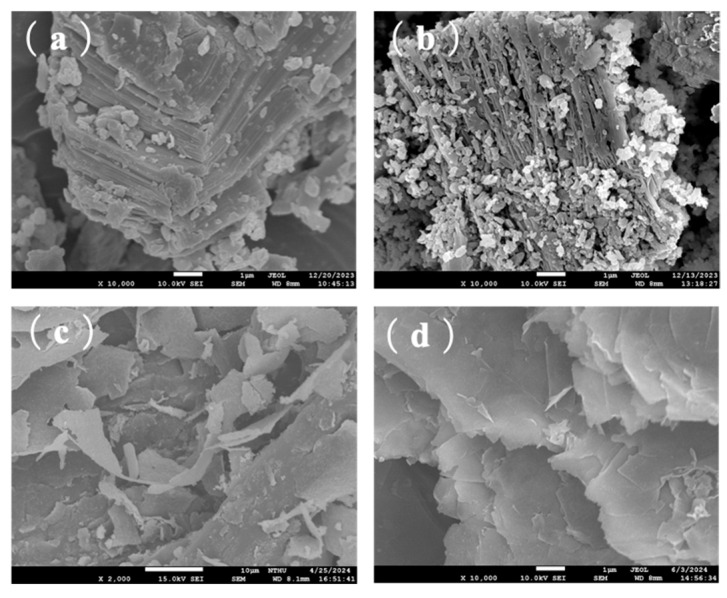
SEM images of (**a**) Ti_3_AlC_2,_ (**b**) Ti_3_C_2_ MXene multilayer, and (**c**,**d**) few-layer of Ti_3_C_2_ MXene nanoflakes at different magnifications.

In this study, the morphology image of Ti_3_AlC_2_ before and after etching was observed by SEM. The Ti_3_AlC_2_ before etching was found to exhibit a dense layer texture structure, as shown in Figure 4a. Post-etching, the material adopts an “accordion” morphology (Figure 4b), with loosely bound multilayered flakes that signpost the onset of delamination. Further ultrasonic shaking and centrifugation yield single-layer MXene nanosheets (Figure 4c) with lateral dimensions up to several microns and a thickness < 1 µm. This high aspect-ratio morphology provides abundant planar surfaces for PANI nucleation and enhances interfacial adhesion in the final composite, which in turn improves both the electrical connectivity and barrier performance in coatings.

### 3.2. Identification of PANI and PA-MX-5

#### 3.2.1. FTIR Analysis of PANI, Ti_3_C_2_ MXene, and PA-MX-5

**Figure 5:**
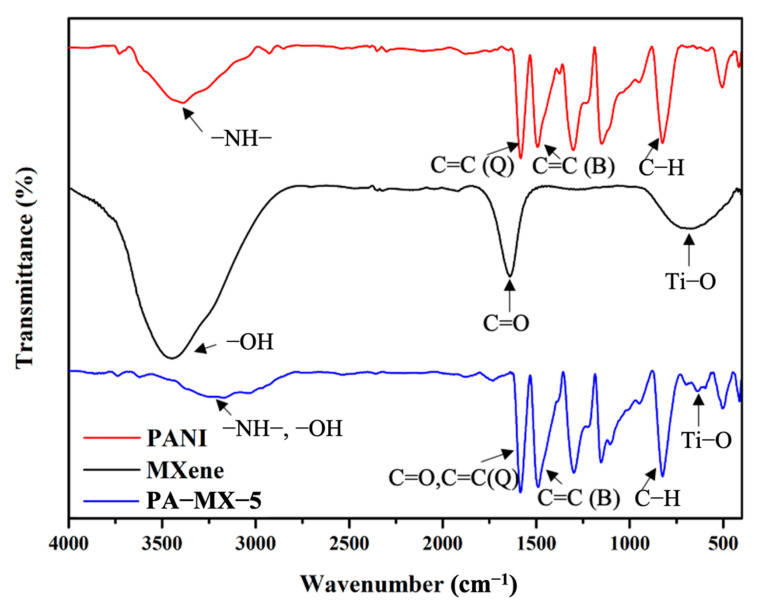
FTIR spectra of PANI, Ti_3_C_2_ MXene, and PA-MX-5.

FTIR spectroscopy provides insight into the chemical interactions and bonding environment in both pure polyaniline (PANI) and its MXene composite, as shown in Figure 5. In the pristine PANI spectrum, strong bands at 1578 cm^−1^ and 1490 cm^−1^ were observed, corresponding to the quinoid (–N=Q=N–) and benzenoid (–N–B–N–) C–N stretching modes, respectively [10]. The peaks at 1298 cm^−1^ and 1247 cm^−1^ arise from C=C stretching vibrations in aromatic and conjugated rings, confirming the extended π-conjugation of emeraldine salt PANI. A broad absorption centered at 3386 cm^−1^ is characteristic of –NH and –NH_2_ stretching, reflecting the presence of a partially protonated backbone. For Ti_3_C_2_ MXene, we detected Ti–O stretching at 674 cm^−1^, C=O stretching at 1640 cm^−1^ (likely from surface terminations), and a broad –OH band at 3450 cm^−1^ from adsorbed water or –OH moieties on the MXene surface [14,15,16,17,18,19,20]. These signals confirm the hydrophilic surface chemistry typical of MILD-etched MXene sheets. In the composite PA-MX-5, all PANI and MXene bands persist, indicating successful coexistence without forming new bulk phases. However, several systematic shifts can be noted: (1) the quinoid C–N band moves from 1578 to 1594 cm^−1^; (2) the benzenoid C–N band from 1490 to 1483 cm^−1^; (3) the aromatic C–H bending from 829 to 821 cm^−1^; and (4) the –NH/–OH envelope broadens and slightly shifts. These red-shifts, combined with peak broadening, are hallmark signatures of hydrogen-bonding and strong interfacial interactions between PANI chains and MXene surfaces. Specifically, hydrogen bonding likely occurs between PANI’s –NH sites and MXene’s –OH groups, as well as electrostatic interactions between protonated PANI segments and negatively charged Ti_3_C_2_ layers. Such interfacial coupling not only stabilizes the composite structure preventing PANI chain aggregation but also promotes enhanced charge-transfer pathways. These stronger electronic interactions are critical for electrocatalytic processes during corrosion protection, as they facilitate rapid electron shuttling through the PANI/MXene network, aiding the formation of passive metal oxide films at the metal interface. FTIR confirms (i) the preservation of PANI’s redox-active backbone, (ii) the retention of MXene’s surface functionalities, and (iii) the emergence of new interfacial interactions: hydrogen bonds and electrostatic coupling that underpin the enhanced electrochemical performance of PA-MX-5.

#### 3.2.2. XRD Analysis of PANI, Ti_3_C_2_ MXene, and PA-MX-5

**Figure 6:**
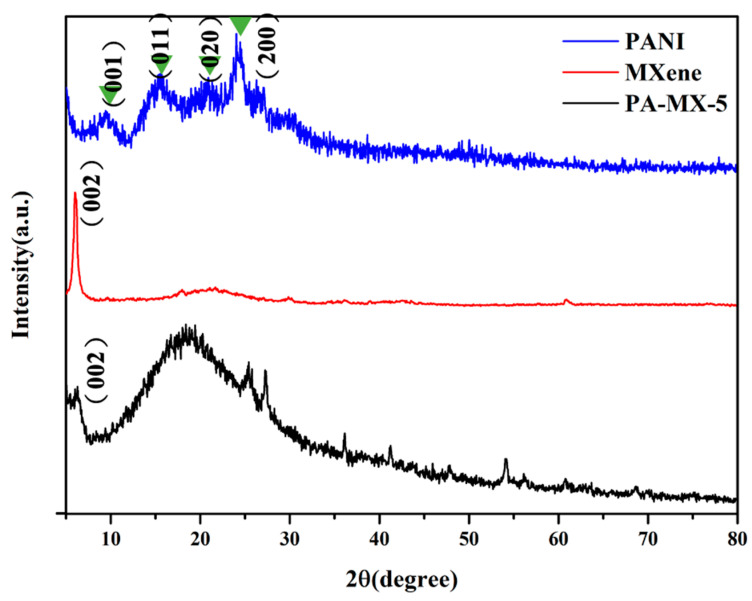
XRD patterns of PANI, Ti_3_C_2_ MXene, and PA-MX-5. Green triangles represent the corresponding peak.

XRD reveals how crystalline ordering evolves upon composite formation (Figure 6). The synthesized pure PANI displays semi-crystalline peaks at 2θ = 8.9°, 15.2°, 20.2°, and 25.2°, corresponding to the (001), (011), (020), and (200) planes of its polymeric structure [37]. These reflections indicate periodic π-stacking and interchain ordering that facilitate electronic delocalization. In PA-MX-5, the PANI peaks remain present and well-defined, with only slight reductions in intensity, evidence that MXene inclusion (5 wt %) does not disrupt the polymer’s semi-crystalline domain. Instead, MXene sheets appear to intercalate between PANI lamellae, preserving the backbone ordering while imparting additional functionality. Notably, the characteristic MXene (002) reflection at 2θ ≈ 6°, strong in isolated Ti_3_C_2_, is absent in the composite. This can be rationalized in two ways: (i). Low MXene loading. At 5 wt %, the sheet population is below the XRD detection threshold, so the (002) peak is too weak to observe. (ii) Encapsulation by PANI. During in-situ polymerization, PANI chains may envelop individual MXene layers, reducing coherent stacking and attenuating the reflection. From an application standpoint, encapsulation is advantageous: it fosters intimate PANI–MXene interfaces critical for charge-transfer coupling. Indeed, later CV measurements show that PA-MX-5 exhibits 1.5–2-fold higher redox currents than pure PANI, consistent with MXene’s role as an electronically conductive scaffold. Moreover, the retention of PANI’s crystallinity ensures that polymer domains maintain pathways for ion transport, while MXene networks supply high-speed electronic conduits. Thus, XRD confirms that PA-MX-5 achieves a synergistic structure: well-ordered PANI domains interpenetrated by dispersed MXene sheets, combining polymer redox activity with 2D electronic percolation.

#### 3.2.3. SEM/TEM Observation of PANI, Ti_3_C_2_ MXene, and PA-MX-5

**Figure 7:**
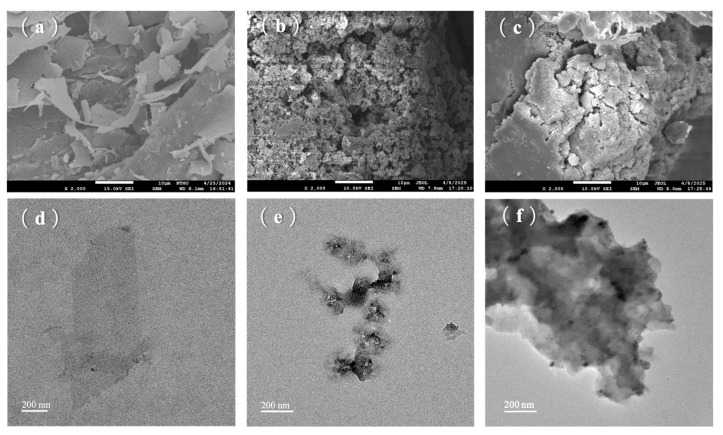
SEM images for (**a**) Ti_3_C_2_ MXene, (**b**) PANI, and (**c**) PA-MX-5, as well as TEM images for (**d**) Ti_3_C_2_ MXene, (**e**) PANI, and (**f**) PA-MX-5.

Electron microscopy elucidates the composite’s morphology at multiple scales. SEM of Ti_3_C_2_ MXene (Figure 7a) shows stacked accordion-like flakes tens of microns across, characteristic of MILD-etched sheets. In contrast, neat PANI (Figure 7b) appears as irregular roughly spherical agglomerates that are hundreds of nanometers to microns in diameter. PA-MX-5 (Figure 7c) exhibits a markedly different morphology: PANI grows conformally on MXene surfaces, yielding a striped fibrous architecture. The PANI fibrils appear aligned along the MXene sheet plane, creating large interfacial contact areas. This intimate wrapping ensures each MXene flake is coated by a PANI shell, minimizing polymer agglomeration and maximizing polymer–sheet interfaces.

TEM further reveals few-layer MXene sheets decorated with PANI fibrils (Figure 7d–f). Here, 3–5 nm wide polymer strands bridging adjacent sheets were observed, establishing a continuous electronic network. The uniform coating indicates that aniline polymerization proceeded preferentially at MXene surfaces, likely nucleated by surface terminations that catalyze oxidative polymerization. These nanoscale observations explain the macroscopic properties: the composite’s enhanced conductivity arises from continuous PANI paths interwoven with MXene electronic backbones, while the tortuous morphology obstructs gas and ion diffusion, improving the barrier performance. Furthermore, the well-dispersed morphology avoids large PANI domains that could crack under stress, enhancing the mechanical robustness of the eventual coating. Overall, SEM/TEM demonstrate that PA-MX-5 achieves a hierarchical architecture of 2D sheets coated with 1D polymer fibrils that underlies its superior multifunctional performance in corrosion protection.

#### 3.2.4. Redox Capability Characterization

##### Electrochemical Cyclic Voltammetry (CV) of PANI and PA-MX-5

**Figure 8:**
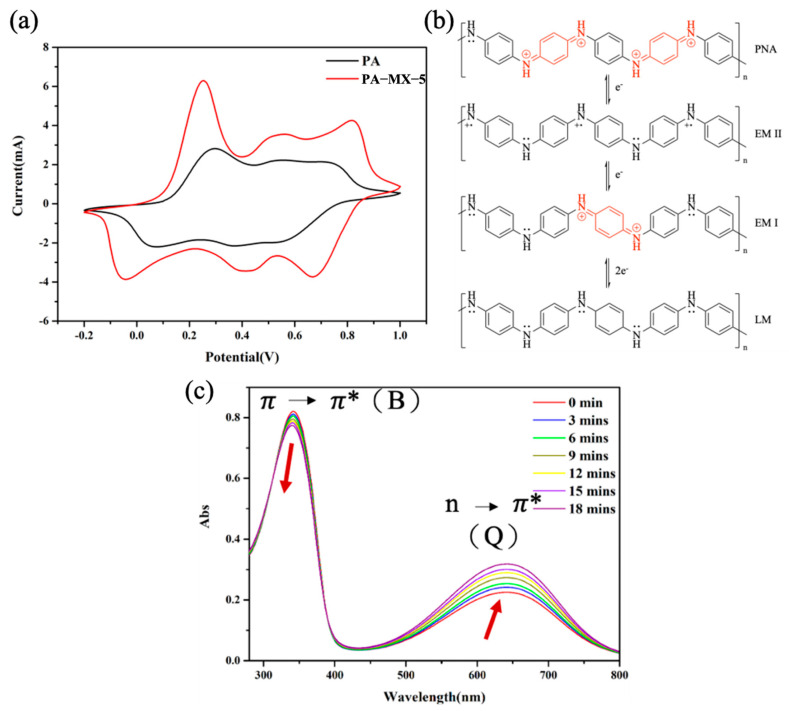
(**a**) Electrochemical cyclic voltammetry curves of PANI and PA-MX-5. (**b**) The proposed oxidation/reduction mechanism of PANI. (**c**) Oxidation of PA-MX-5 by the step-wise introduction of ammonium persulfate monitored by the UV-Visible absorption spectroscopy.

CV was conducted using a standard three-electrode system, with SCE as the reference electrode, at a scan rate of 100 mV/s, to evaluate the redox capability of PA. Under these conditions, three pairs of reversible redox peaks were observed in the CV curves of PANI, as shown in Figure 8a, with oxidation peaks at 0.31, 0.56, and 0.75 V, corresponding to the transitions between the fully reduced leucoemeraldine (LM) state, the emeraldine I (EM I) state, and the fully oxidized pernigraniline (PNA) state. The corresponding reduction peaks were observed at 0.55, 0.39, and 0.07 V, respectively, indicating the reversibility of PANI during the redox process and confirming the successful synthesis of PANI with excellent electrochemical activity.

On the other hand, PA-MX-5 exhibited a significantly higher redox current than that of neat PANI, as shown in Figure 8. This is attributed to the disruption of nanoparticle agglomeration in PANI, which exposes more electroactive sites on the surface. Like PANI, PA-MX-5 also displayed three pairs of redox peaks. Compared with Figure 8, the first redox pair appeared at the potential of 0.25 V and 0.05 V (LM → EM I), the second redox pair appeared at the potential 0.54 V and 0.42 V (EM I → EM II), and the third redox pair appeared at the potential of 0.82 V and 0.67 V (EM II → PNA). The larger peak currents of PA-MX-5 exhibited an obviously higher redox capacity as compared to that of neat PANI, indicating that the good dispersion of the small loading of Ti_3_C_2_ MXene nanosheets may effectively promote the redox capability of PANI. The proposed mechanism for the oxidation/reduction of PANI is shown in Figure 8b.

##### UV-Visible Absorption Spectroscopy of PA-MX-5

UV-Visible spectroscopy was used to track PANI’s electronic transitions under stepwise oxidation. In the fully reduced leucoemeraldine state, PANI exhibits a π → π* transition band near 350 nm, while the oxidized emeraldine/quinoid form shows an n → π* band around 650 nm [38]. Figure 8c shows that, as the APS oxidant is incrementally added to PA-MX-5 suspensions, the π → π* band intensity diminishes, while the n→π* band grows consistent with the conversion to higher oxidation states. A slight blue-shift (350 → 335 nm) of the π → π* peak and a red-shift (640 → 645 nm) of the n → π* peak were observed. These wavelength shifts indicate that MXene’s surface charges and hydrogen bonding influence PANI chain conformation, slightly altering the energy levels of the π and n orbitals. Specifically, electron donation from MXene to PANI’s backbone may stabilize the quinoid structures, lowering the n → π* transition energy and causing the red-shift. Additionally, hydrogen bonds can restrict polymer chain planarity, leading to the π → π* blue-shift. These subtle spectral changes confirm strong PANI–MXene electronic coupling, which is key to the composite’s enhanced redox reversibility and stability, as corroborated by CV results. Thus, UV-Vis spectroscopy not only tracks oxidation progression but also reveals how MXene modulates PANI’s electronic structure, enabling tunable redox behavior crucial for self-healing passive film generation on metal substrates.

#### 3.2.5. Oxygen Gas Permeability Analysis of PANI and Ti_3_C_2_ MXene-Based PANI Composite Free-Standing Membranes

**Figure 9:**
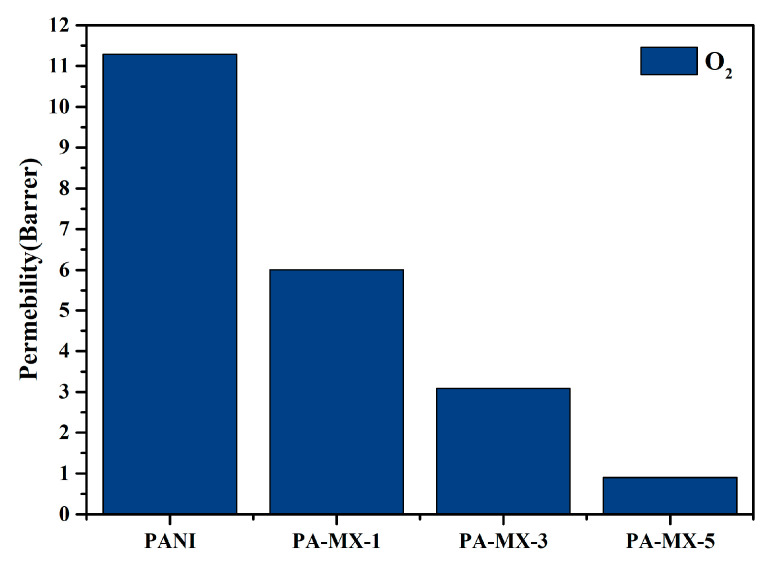
Oxygen gas permeability analysis (GPA) of PANI, PA-MX-1, PA-MX-3, and PA-MX-5.

Gas permeability measurements quantify the barrier efficacy of polymer films. Neat PANI membranes (~60 µm thick) exhibit an oxygen permeability of 11.29 Barrer (Figure 9). Introducing Ti_3_C_2_ MXene at 1 wt % reduces the permeability to 6.00 Barrer, a 47% decrease, while 3 wt % and 5 wt % loadings further lower the values to 3.08 Barrer and 0.90 Barrer, respectively. This dramatic improvement arises from MXene’s high aspect ratio: dispersed sheets create tortuous diffusion pathways that drastically increase the effective path length for gas molecules [16,17,18,19,20]. At the molecular level, oxygen molecules must navigate around impermeable MXene barriers, reducing flux. Even a small loading of 1 wt % yields significant path tortuosity; beyond 3 wt %, MXene sheets begin to overlap, forming near-continuous barriers that approach impermeability. For anticorrosion coatings, reduced O_2_ and H_2_O permeability is critical: it limits oxidant access to the underlying steel, slowing corrosion reactions.

#### 3.2.6. XRD Characterization of Densely Passive Metal Oxide Layer Induced by the Electro-Catalyzed Capability of PA-MX-5

**Figure 10:**
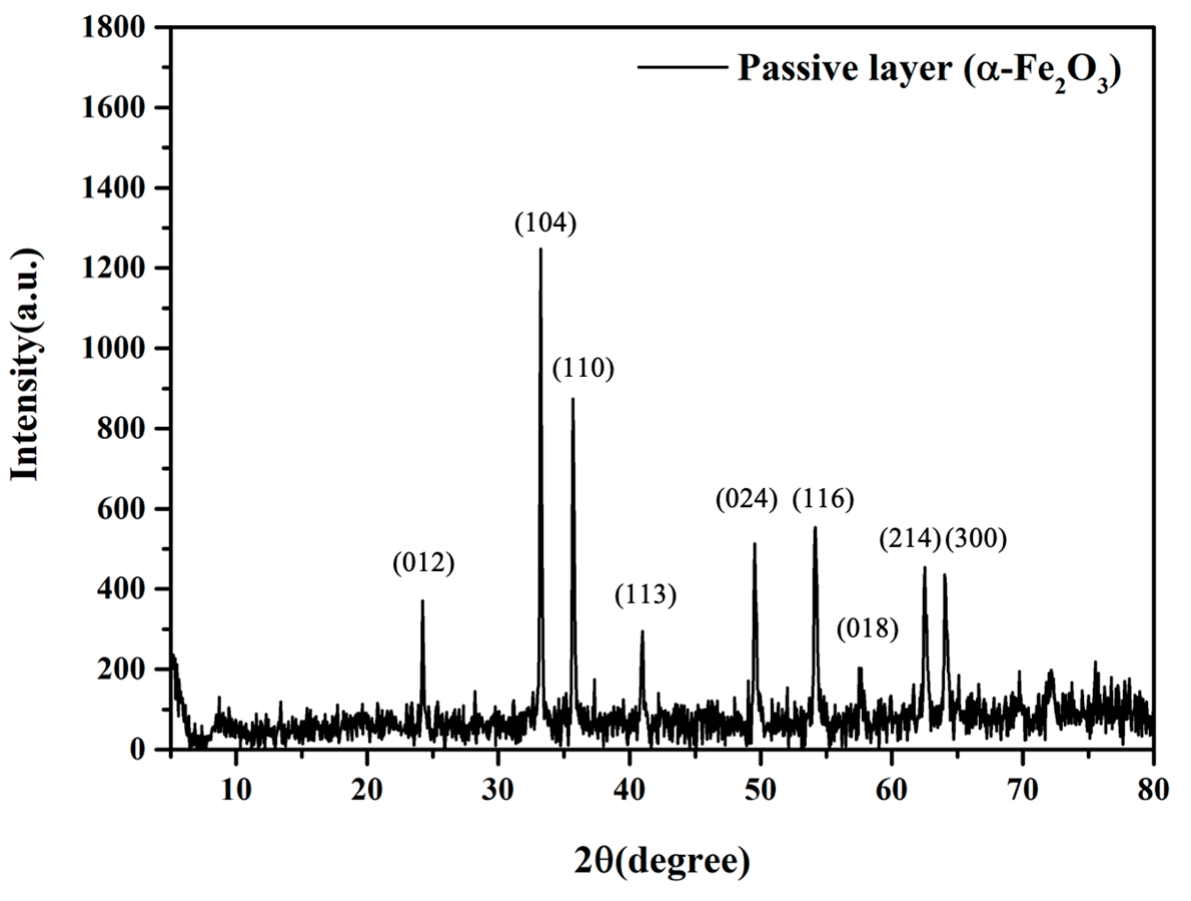
Formation of densely passive metal oxide layers of Fe_2_O_3_ induced by the electrocatalytic capability of PANI.

The CRS electrode coated with as-prepared PA-MX-5 coating was maintained for 30 days for the formation of densely passive metal oxide layers induced from the electro-catalyzing capability of PA-MX-5. The as-prepared PA-MX-5 coating was found to exhibit a much stronger electro-catalyzing capability than that of neat PANI, as shown by the electrochemical CV data in the previous section. In Figure 10, the XRD pattern of passive metal oxide layers was found to form on the surface of metallic CRS electrode, which is similar to the previous report in the literature on the formation of passive metal oxide layers induced from the electro-catalyzing capability of PANI [39].

### 3.3. Structural Characterization of Bio-PANI and Bio-PA-MX-5

#### 3.3.1. Surface Morphology Observed by Scanning Electron Microscopy (SEM)

**Figure 11:**
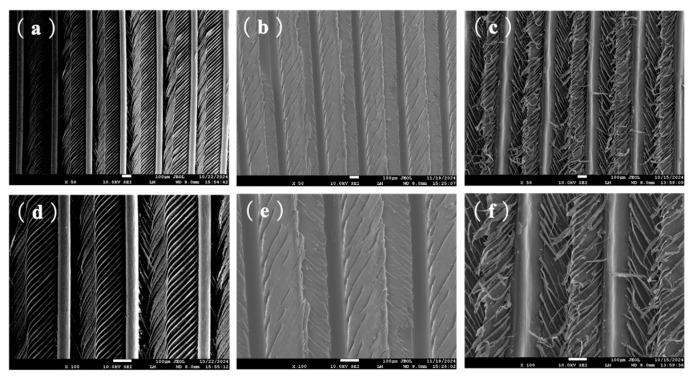
SEM images for (**a**,**d**) the surface morphology of natural macaw feathers at different magnifications, (**b**,**e**) the surface morphology of PDMS negative soft template with biomimetic structure at different magnifications, and (**c**,**f**) the artificial surface morphology of PA-MX-5 coating with 3D biomimetic structure of macaw feather.

To investigate whether the nano-casting technique faithfully replicates the intricate architecture of natural macaw feathers, SEM was performed on (i) the original feather, (ii) the PDMS negative template, and (iii) the Bio-PA-MX-5 composite. As shown in Figure 11a,d, natural macaw feathers possess a hierarchical arrangement: primary barbs (10–20 µm diameter) bearing secondary barbules (1–2 µm), which branch further into hooklets that interlock to form the vane. This multiscale anisotropic geometry underlies feathers’ remarkable hydrophobicity and mechanical resilience [26,27]. The PDMS negative template (Figure 11b,e) captures these features with high fidelity. Primary barbs appear as micrometer-scale ridges in the elastomer, while deep recesses mimic the barbule network. The smooth PDMS surface inverts the feather topography, creating a robust soft mold that can tolerate repeated casting cycles without significant wear or distortion. Eventually, with the Bio-PA-MX-5 coating, as shown in Figure 11c,f, the PANI/MXene composite inherits the multi-tiered structure: protruding ridges (~10 µm) alternate with valleys containing micro-scale grooves (~1–2 µm). Importantly, the composite surface shows no signs of template collapse or polymer overfilling; the barbule-like grooves remain open and well-defined. This confirms that the PDMS mold does not indent or smear the coating during de-molding. Replicating this complex geometry is critical because it dramatically increases the apparent surface roughness at both the micro- and nanoscale. Thus, the SEM results unequivocally demonstrate that nano-casting into PDMS accurately transfers hierarchical feather structures into the composite, a prerequisite for achieving superhydrophobicity and enhanced corrosion resistance.

#### 3.3.2. Water Contact Angle (CA)

**Figure 12:**
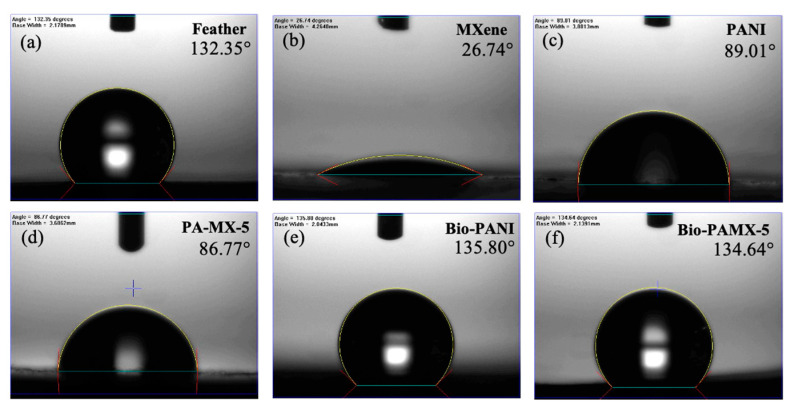
Contact angles of water droplets on the surface of (**a**) natural macaw feather, (**b**) PANI, (**c**) PA-MX-5, (**d**) Ti_3_C_2_ MXene, (**e**) Bio-PANI, and (**f**) Bio-PA-MX-5.

In this study, the CA measurement of water droplets was used to evaluate the wettability for the surface of the as-prepared distinctive materials. The CA measurement of water droplets was performed by placing 10 μL of deionized water droplet onto the surface of samples. The CA value of the natural macaw feather and neat PANI was found to be ~132° and ~90°, respectively, as shown in Figure 12a,b. The CA value of neat Ti_3_C_2_ MXene was found to be ~27°, indicating that the Ti_3_C_2_ MXene is quite hydrophilic, as shown in Figure 12c. By dispersing the small amount of Ti_3_C_2_ MXene nanosheets in PANI, the CA value of PA-MX-5 was found to be ~87°, which is slightly lower than that of neat PANI, reflecting the dispersion of hydrophilic MXene nanosheets into the PANI matrix. After treating PANI with the nano-casting technique, the CA for the surface of the PANI and PA-MX-5 coating with 3D biomimetic surface structure (denoted Bio-PANI and Bio-PA-MX-5) was found to be increased to ~136° and 135°, respectively, indicating that the PANI with a biomimetic structure may promote an increase in the contact angle of ~46–48°, leading to a more hydrophobic material surface. Nonetheless, the CA value of the artificial biomimetic coatings was found to quite similar to that of natural macaw feather. Hydrophobicity is pivotal for anticorrosion: water droplets readily roll off, carrying away salts and contaminants, and the trapped air layer further impedes electrolyte contact. Therefore, the nano-casting approach efficiently endows the polymeric coating with feather-inspired hydrophobicity.

#### 3.3.3. BET Study

**Figure 13:**
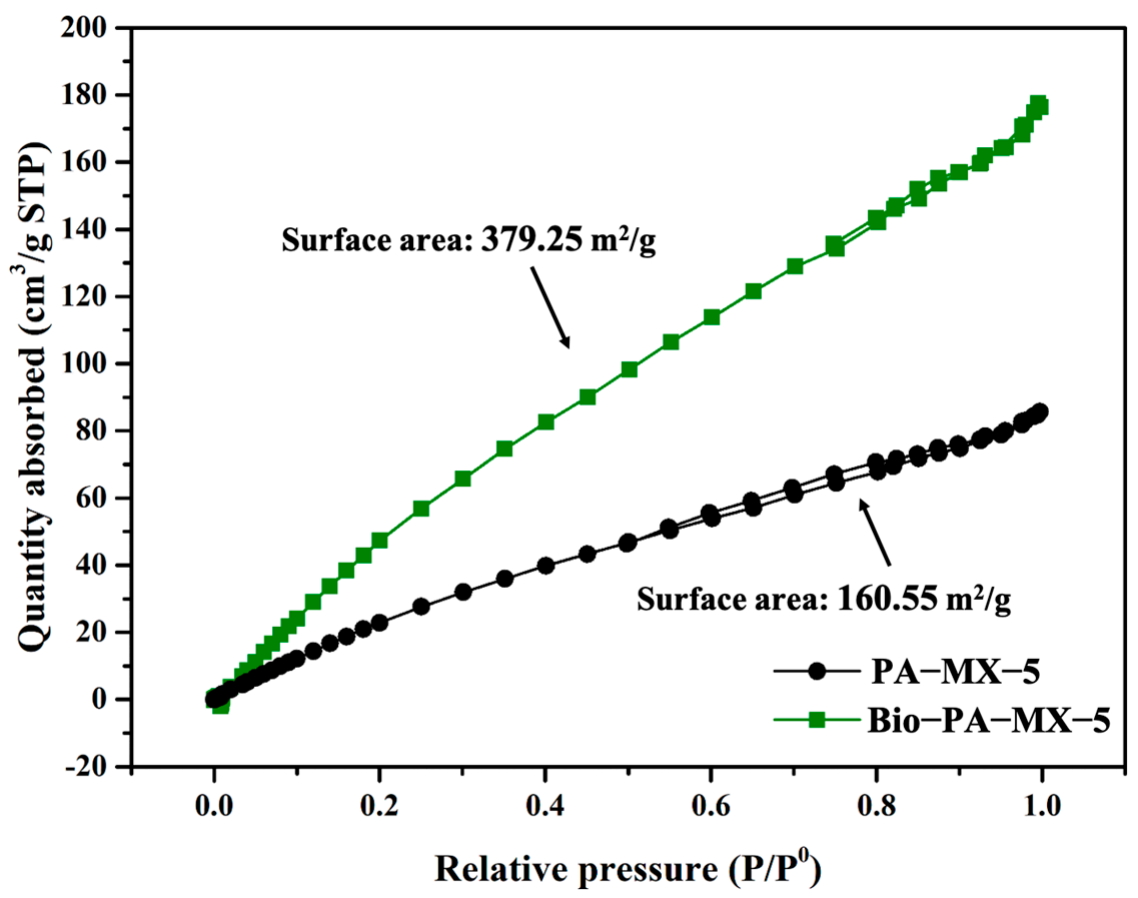
Adsorption isotherm curve of PA-MX-5 and Bio-PA-MX-5.

BET analysis probes a material’s specific surface area, which in porous or rough coatings influences both the barrier performance and catalytic activity. Nitrogen adsorption–desorption isotherms for PA-MX-5 and Bio-PA-MX-5 (Figure 13) exhibit Type II behavior, characteristic of nonporous or macroporous materials with unrestricted multilayer adsorption. At low relative pressure, Bio-PA-MX-5 with a biomimetic surface structure was found to exhibit a significantly higher adsorption capacity than PA-MX-5. At high pressure, Bio-PA-MX-5 exhibited a greater nitrogen uptake, providing more space for gas adsorption. Quantitative analysis yielded a specific surface area of 160 m^2^/g for PA-MX-5 and 380 m^2^/g for Bio-PA-MX-5, representing a 2.4-fold increase due purely to the introduction of the hierarchical feather-inspired topography. BET quantification demonstrates that nano-casting the macaw feather geometry onto the PANI/MXene composite nearly tripled the accessible surface area and significantly enhanced the total pore volume, purely through topographical modification (no chemical additives).

### 3.4. Corrosion Protection Determined by Electro-Chemical Measurements

#### 3.4.1. MXene-Based PANI Composites with Distinctive Loading of Ti_3_C_2_ Nanosheets

##### Tafel Test

**Figure 14:**
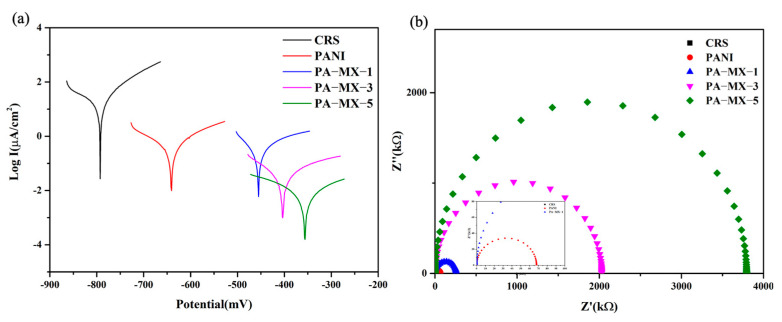
(**a**) Tafel plots and (**b**) Nyquist plots for raw CRS electrode and CRS electrode coated with PANI and its derived MXene-based composites.

The corrosion protection performance of coatings was determined by Tafel plots of the CRS electrode and the CRS electrode coated with distinctive samples with ~80 μm of coating thickness in 3.5 wt % of NaCl (aq). As shown in Figure 14a, increasing the Ti_3_C_2_ MXene content shifts the corrosion potential (E_corr_) in the positive direction, from −588 mV for PANI up to −356 mV for PA-MX-5, indicating more noble behavior. Correspondingly, the corrosion current density (I_corr_) decreases markedly with higher MXene loading: 1.09 μA/cm^2^ for PANI, 4.73 × 10^−1^ μA/cm^2^ for PA-MX-1, 6.52 × 10^−2^ μA/cm^2^ for PA-MX-3, and 3.52 × 10^−2^ μA/cm^2^ for PA-MX-5. Since a lower I_corr_ directly correlates with a reduced corrosion rate, these results demonstrate that higher MXene incorporation significantly enhances the coating’s anticorrosion performance. All quantitative values are compiled in Table 1 for reference.

##### Nyquist Plots Determined by Impedance Spectroscopy (EIS)

Moreover, the corrosion protection of coatings determined by EIS measurements were also performed through immersing the CRS electrode and the CRS electrode coated with distinctive samples with ~80 μm of coating thickness in 3.5 wt % of NaCl (aq). The impedance of the MXene-based PANI composites was found to be increased with the increasing loading of Ti_3_C_2_ MXene, as shown in Figure 14b. For example, the impedance of the as-prepared coatings was found to exhibit an increasing trend as follows: PANI (67 kΩ) < PA-MX-1 (258 kΩ) < PA-MX-3 (2029 kΩ) < PA-MX-5 (3794 kΩ). Therefore, in this study, the largest impedance value of 3794 kΩ can be found in the coating of PA-MX-5. All the quantitative data for the impedance are also summarized in Table 1. The trend for the Nyquist plots of EIS for the corrosion protection of coatings were consistent with the previous results obtained from the Tafel plots. Therefore, the PA-MX-5 coating was selected as the candidate for the subsequent corrosion protection study for biomimetic processing through the nano-casting technique.

#### 3.4.2. Corrosion Protection of Coatings with Biomimetic Surface Structure Prepared from the Nano-Casting Technique

##### Tafel Test

**Figure 15:**
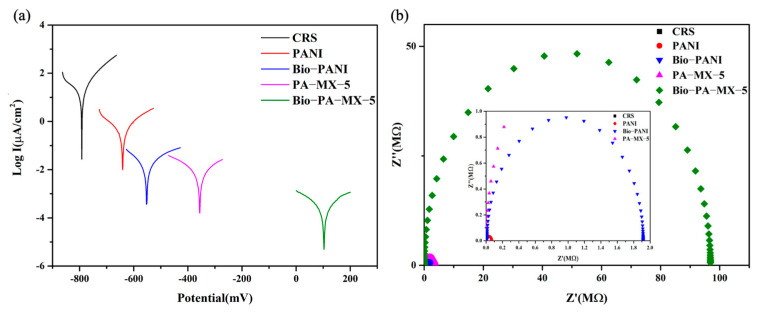
(**a**) Tafel plots and (**b**) Nyquist plots for CRS electrode and CRS electrode coated with four distinctive samples of PANI, Bio-PANI, PA-MX-5, and Bio-PA-MX-5.

The corrosion protection performance of coatings was determined by Tafel plots of the CRS electrode and the CRS electrode coated with coatings without/with the biomimetic surface structure in 3.5 wt % of NaCl (aq). The Tafel plots of coatings with the biomimetic surface structure was also found to exhibit better corrosion protection as compared to that of coating without biomimetic surface structure, as shown in Figure 15b. Introducing the feather-inspired topography shifts the E_corr_ to more positive values: from −641 mV for PANI to −552 mV for Bio-PANI and from −356 mV for PA-MX-5 to +103.6 mV for Bio-PA-MX-5. More importantly, the I_corr_ decreases substantially upon biomimetic structuring, dropping from 1.09 μA/cm^2^ for PANI to 4.76 × 10^−2^ μA/cm^2^ for Bio-PANI and from 3.52 × 10^−2^ μA/cm^2^ for PA-MX-5 to 7.22 × 10^−4^ μA/cm^2^ for Bio-PA-MX-5. Since a lower I_corr_ corresponds to a lower corrosion rate, these results confirm that the biomimetic surface greatly enhances the coating’s resistance to corrosion.

##### Nyquist Plots Determined by Impedance Spectroscopy (EIS)

Moreover, the EIS tests conducted in 3.5 wt % NaCl (aq) were also employed to evaluate the corrosion resistance of PA-MX-5 without/with the 3D biomimetic surface structure of natural macaw feather, as shown in Figure 15. From Figure 15b, the Nyquist plots of both the PANI and PA-MX-5 with biomimetic surface structure exhibited a larger semicircle as compared to that of corresponding coating without the biomimetic structure. For example, the Z″ value of the Bio-PA-MX-5 coating was found to be ~96,875 kΩ, which is ~50 times higher than that of Bio-PANI (1915 kΩ) and ~25 times higher than that of PA-MX-5 (3794 kΩ). The trend for the Nyquist plots of EIS for the corrosion protection of coatings were consistent with the previous results obtained from the Tafel plots. The EIS data in Bode (Figure S1) are presented to illustrate the full impedance response across our applied frequency sweep. The impedance magnitude curves exhibit a distinct high-frequency plateau that aligns with the solution resistance extracted from Nyquist analysis, while the gradual roll-off at lower frequencies reflects the combined effects of charge-transfer and polymer-film resistance. The corresponding phase-angle spectra show a single broad maximum, confirming the dominance of a single time-constant process consistent with our equivalent-circuit model. This Bode representation allows readers to visually assess the transition from ohmic to interfacial behavior without requiring explicit frequency endpoint values.

### 3.5. Mechanism for Enhancement of Anticorrosion of CRS Electrode Coated with Ti_3_C_2_ MXene-Based PANI Composite with Artificial Biomimetic Surface Structure

**Figure 16:**
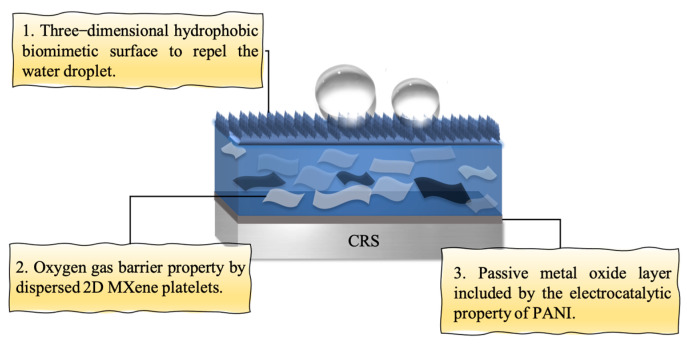
Three distinctive possible corrosion protection mechanisms were integrated to support the enhancement of anticorrosion for the CRS electrode coated with the electroactive PANI/Ti_3_C_2_ MXene composite with the biomimetic 3D surface structure of a macaw feather.

The superior corrosion resistance of the Bio-PA-MX-5 coating arises from the synergistic integration of three protective mechanisms (Figure 16): (i) PANI’s electrocatalytic formation of a dense passive oxide, (ii) MXene’s creation of a tortuous gas barrier, and (iii) biomimetic surface structuring that dramatically increases hydrophobicity.

(i) Electrocatalytic passive-film formation by PANI.

Polyaniline’s reversible redox chemistry enables it to mediate oxidation of Fe^2+^ ions at the steel interface. Under anodic polarization or even at open-circuit potential, PANI transfers electrons to molecular oxygen, generating local oxidizing equivalents that convert nascent iron into a uniform Fe_2_O_3_/Fe_3_O_4_ passive layer. XRD after 30 days shows strong crystalline Fe_2_O_3_ reflections only on PA-MX-5–treated electrodes (Figure 9), indicating a thicker more ordered oxide film than with PANI alone. This electrocatalysis both consumes corrosive species and builds a mechanically robust barrier that tightly adheres to the substrate, effectively blocking further metal dissolution.

(ii) Gas-permeability barrier from 2D MXene nanosheets.

The incorporation of Ti_3_C_2_ MXene nanosheets into the PANI matrix creates overlapping high-aspect-ratio platelets that force gas molecules—O_2_, H_2_O, Cl^−^ ions—to navigate a labyrinthine path. GPA measurements show the oxygen permeability dropping from 11.3 Barrer (PANI) to 0.90 Barrer (PA-MX-5) at 5 wt % MXene (Figure 8). By limiting the diffusion of oxidants and moisture to the steel surface, MXene transforms the composite from a simple polymer film into a nearly impermeable barrier. This physical obstruction works in concert with PANI’s electrocatalysis: the passive film formed is shielded from aggressive species, enhancing its longevity.

(iii) Hydrophobicity via biomimetic surface structuring.

Nano-casting the surface of macaw feathers imparts a hierarchical dual roughness—micron-scale ridges and submicron grooves—onto the composite. The water CA measurements jump by ~46–48° after structuring (from ~87° to ~135°), entering the hydrophobic regime (Figure 11). On such surfaces, water droplets bead up and roll off, carrying away salt deposits and preventing prolonged electrolyte contact. The trapped air layer beneath droplets further reduces liquid–solid contact fraction, as described by the Cassie–Baxter equation, minimizing the capillary-driven wetting of defects. This topographical barrier complements the chemical and physical protections by ensuring rapid droplet removal and reducing standing corrosive solution on the coating.

Together, these three mechanisms—electrocatalytic passivation, MXene-enhanced tortuosity, and feather-mimetic hydrophobicity—act in concert to deliver outstanding long-term corrosion protection for steel substrates under harsh saline conditions.

Additional WCA, SEM, and BET measurements on PA-MX-5 samples were conducted after the electrochemical tests. As shown in Figure S2, the contact angle remained within ±2° of the as-prepared value, and the BET surface area changed by <5%, indicating minimal surface degradation. The SEM—observed morphology indicates that its hierarchical structure and superhydrophobicity remain stable under oxidative conditions and thus confer long-term durability in anticorrosion applications.

## 4. Conclusions

This study aimed to prepare effective corrosion protection coatings by integrating three distinctive anticorrosion methodologies. First, Ti_3_C_2_ MXene nanosheets were successfully synthesized by treating the Ti_3_AlC_2_ with the MILD method, followed by characterization by XRD and SEM. Subsequently, the Ti_3_C_2_ MXene-based PANI composites were prepared by oxidative polymerization of aniline monomers in the presence of 1, 3, and 5 wt% of Ti_3_C_2_ MXene nanosheets with APS as the oxidant (denoted by PA-MX-1, PA-MX-3, and PA-MX-5), followed by systematic characterization through FTIR, XRD, SEM, TEM, CV, and UV-Visible. Moreover, the artificial coating with the biomimetic surface structure of a natural macaw feather was prepared by the nano-casting technique, followed by characterization through SEM, CA, and BET. The corrosion protection of coatings with the distinctive loading of Ti_3_C_2_ MXene nanosheets and with the biomimetic surface structure was investigated by electrochemical corrosion measurements such as Tafel plots and Nyquist plots. The mechanism for explaining the enhancement of anticorrosion performance of the as-prepared Ti_3_C_2_ MXene-based PANI composite coating with an artificial biomimetic surface structure may be attributed to the integration of three possible mechanisms: (i) the electro-catalyzing capability of PANI may induce the formation of a densely passive metal oxide layer, (ii) the good dispersion of Ti_3_C_2_ MXene nanosheets in PANI may boost the oxygen gas barrier property of the corresponding composite films/membranes, and (iii) the coating with the biomimetic surface structure exhibited higher hydrophobicity to repel water molecules.

## Figures and Tables

**Table 1:** Electrochemical corrosion measurement and oxygen permeability of raw CRS electrode and CRS coated with distinctive coatings immersed 3.5 wt % of NaCl aqueous solution.

*Sample Code*	*Composition Ratio*		*Electrochemical Corrosion Measurement*			*P_EF_* (%)	*Z*′ (kΩ)	*Oxygen Permeability* (Barrer)	*Thickness* (µm)
	*Aniline*	*MXene*	*E_corr_* (mV)	*R_p_* (kΩcm^2^)	*I_corr_* (µA/cm^2^)				
*CRS*	-	-	−791.9	8.71 × 10^−1^	5.74 × 10	-	0.0259	-	-
*PANI*	100	0	−588.3	4.63 × 10^1^	1.09	98.10	67	11.2910	82 ± 2
*PA-MX-1*	100	1	−455.2	5.53 × 10^1^	4.73 × 10^−1^	99.17	258	6.0034	80 ± 2
*PA-MX-3*	100	3	−402.4	5.86 × 10^2^	6.52 × 10^−2^	99.88	2029	3.0795	81 ± 2
*PA-MX-5*	100	5	−356.0	3.19 × 10^3^	3.52 × 10^−2^	99.93	3794	0.8978	80 ± 1
*Bio-PANI*	100	0	−552.0	1.36 × 10^2^	4.76 × 10^−2^	99.91	1915	-	85 ± 5
*Bio-PA-MX-5*	100	5	103.6	8.65 × 10^4^	7.22 × 10^−4^	99.998	96875	-	85 ± 6

## Data Availability

The data that support the findings of this study are available from the corresponding author, but restrictions apply to the availability of these data, which were used under license for the current study, and so are not publicly available. The data are, however, available from the authors upon reasonable request and with permission of the funding party, Ministry of Science and Technology, Taiwan.

## Supplementary Materials

The following supporting information can be downloaded at: https://www.mdpi.com/article/10.3390/biomimetics10070465/s1; Figure S1: Plots of Bode for raw CRS electrode and CRS electrode coated with (a) PANI and its derivating MXene-based composites; (b) PANI, Bio-PANI, PA-MX-5, and Bio-PA-MX-5; Figure S2: Plots of WCA, SEM and BET before and after oxidation of Bio-PA-MX-5.
