# Supplementary material for: Polyaniline/Ti3C2 MXene Composites with Artificial 3D Biomimetic Surface Structure of Natural Macaw Feather Applied for Anticorrosion Coatings
Source: Biomimetics (Basel). 2025 Jul 15;10(7):465. doi: 10.3390/biomimetics10070465 (PMC12292397; doi:10.3390/biomimetics10070465)
Supplement: Supplementary file 1 [file biomimetics-10-00465-s001.zip › biomimetics-3661933-supplementary.pdf]

Supplementary material

Polyaniline/Ti_3C_2 MXene Composites with Artificial 3D Biomimetic Surface Structure of Natural Macaw Feather Applied for Anticorrosion Coatings

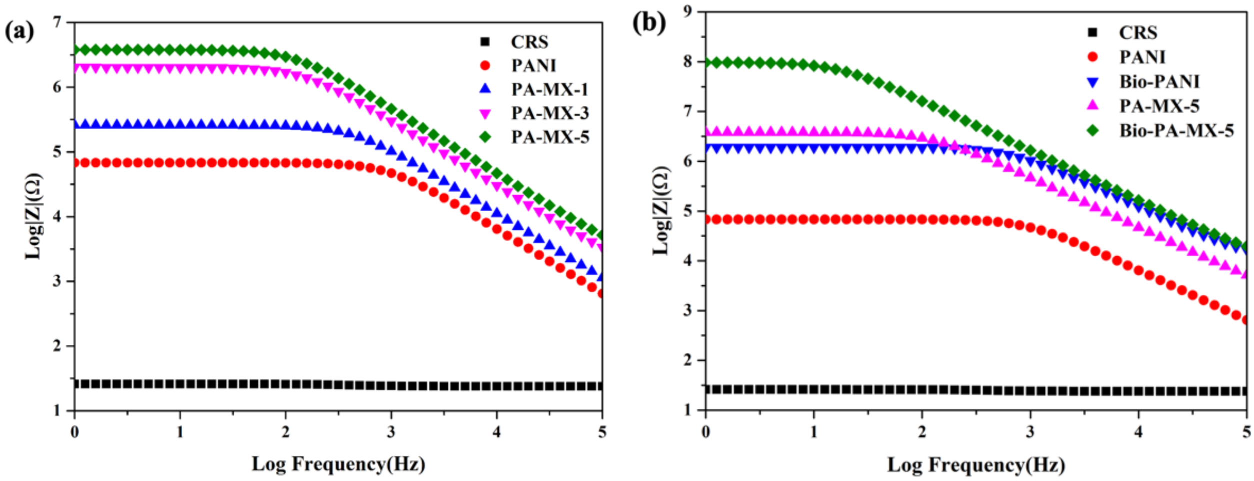

Figure S1. Plots of Bode for raw CRS electrode and CRS electrode coated with(a) PANI and its derivating MXene-based composites.(b) PANI, Bio-PANI, PA-MX-5, and Bio-PA-MX-5.

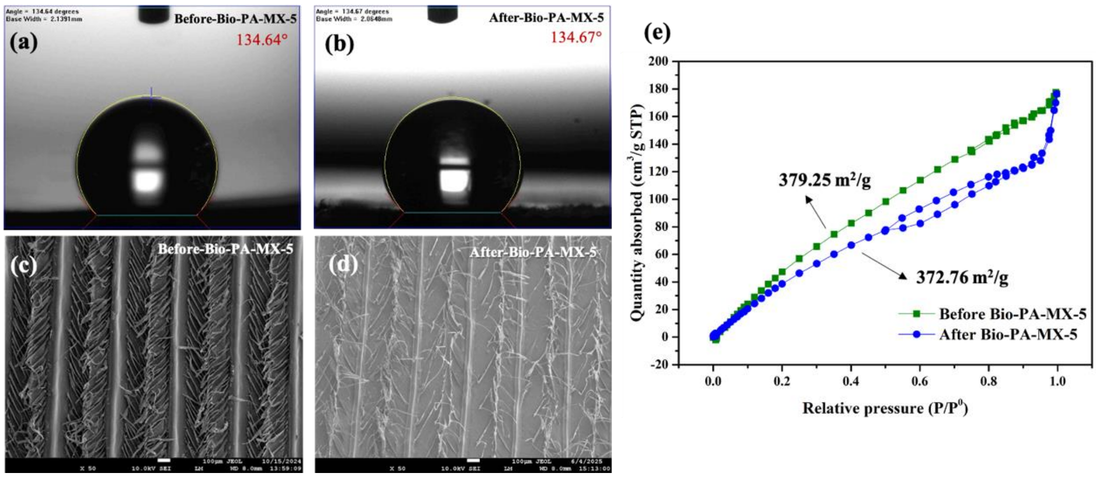

Figure S2. Plots of WCA, SEM and BET before and after oxidation of Bio-PA-MX-5.
